# Temperature-Decoupled Single-Crystal MgO Fiber-Optic Fabry–Perot Vibration Sensor Based on MEMS Technology for Harsh Environments

**DOI:** 10.3390/mi15050616

**Published:** 2024-05-01

**Authors:** Chengxin Su, Pinggang Jia, Aihao Zhao, Jiacheng Tu, Jia Liu, Qianyu Ren, Jijun Xiong

**Affiliations:** State Key Laboratory of Dynamic Measurement Technology, North University of China, Taiyuan 030051, China; suchengxin08@163.com (C.S.); zah5401@163.com (A.Z.); s202206077@st.nuc.edu.cn (J.T.); jialiu@nuc.edu.cn (J.L.); renqianyu1315@126.com (Q.R.); xiongjijun@nuc.edu.cn (J.X.)

**Keywords:** fiber-optic, single-crystal MgO, vibration measurement, temperature decoupling

## Abstract

A high-temperature-resistance single-crystal magnesium oxide (MgO) extrinsic Fabry–Perot (FP) interferometer (EFPI) fiber-optic vibration sensor is proposed and experimentally demonstrated at 1000 °C. Due to the excellent thermal properties (melting point > 2800 °C) and optical properties (transmittance ≥ 90%), MgO is chosen as the ideal material to be placed in the high-temperature testing area. The combination of wet chemical etching and direct bonding is used to construct an all-MgO sensor head, which is favorable to reduce the temperature gradient inside the sensor structure and avoid sensor failure. A temperature decoupling method is proposed to eliminate the cross-sensitivity between temperature and vibration, improving the accuracy of vibration detection. The experimental results show that the sensor is stable at 20–1000 °C and 2–20 g, with a sensitivity of 0.0073 rad (20 °C). The maximum nonlinearity error of the vibration sensor measurement after temperature decoupling is 1.17%. The sensor with a high temperature resistance and outstanding dynamic performance has the potential for applications in testing aero-engines and gas turbine engines.

## 1. Introduction

Vibration is a key parameter for safety monitoring in aero-engine testing and an important reference for analyzing faults [1,2,3]. As the thrust-to-weight ratio of the aero-engine increases, the combustion chamber developments lean toward a high-temperature rise and high heat capacity. The operating environment of sensors has become harsher [4,5,6]. Traditional high-temperature vibration sensors mainly utilize the piezoelectric effect of piezoelectric materials, whose maximum operating temperature is usually lower than 800 °C because of the limitation of the Curie point, and the measurement accuracy is affected by the temperature [7,8,9,10]. Compared with piezoelectric sensors, fiber-optic vibration sensors are widely used in vibration measurement due to the advantages of light weight, large dynamic range, and anti-interference. The characteristics of miniaturization, high robustness, and high temperature resistance have gradually become the development direction of fiber-optic vibration sensors [11,12,13,14].

The existing fiber-optic vibration sensors include intensity modulation, wavelength modulation, and phase modulation types. The demodulation accuracy of intensity-modulated fiber-optic vibration sensors is affected by light source fluctuations and transmission losses [15,16]. Wavelength-modulated fiber-optic vibration sensors generally use fiber Bragg grating (FBG) as sensing components, and their sensitivity and dynamic testing range are limited by the grating linewidth [17,18]. In comparison, the phase modulation type utilizes the phase change of optical signals to achieve vibration measurement, which has the advantages of high accuracy and sensitivity [19,20]. At present, fiber-optic vibration sensors based on Mach–Zender interference [21], Michelson interference [22], and Fabry–Perot (FP) interference [23] have been developed. The FP interferometric fiber-optic sensors have received widespread attention and research in the field of vibration measurement due to their simple structure, easy production, and high resolution. Nieva et al. [24] developed an FP interferometric high-temperature vibration sensor based on micro electro mechanical systems (MEMS) technology for spatial light transmission. The substrate and cantilever beam were placed in parallel to form an FP interferometer with a high acceleration sensitivity, and it can work at 600 °C. The single cantilever beam structure used in the sensor ensures a minimum detectable displacement of approximately 0.139 nm Hz^−1/2^. Ran et al. [25] used excimer lasers to process FP cavities at the end faces of large cladding fibers for vibration measurement, achieving the thermal stress matching manufacturing of sensors. The sensor had an average sensitivity of 1.76 rad/g in the 0–100 Hz range. Cui et al. [26] proposed a high-temperature vibration sensor based on sapphire fiber. The vibration diaphragm was a support beam structure made of single-sided polished sapphire chips by femtosecond laser etching. The sensor was subjected to vibration testing at 25 °C, 300 °C, and 600 °C, and the output error did not exceed 1% of the full range. The resonant frequency of the sensor was 2700 Hz, and the sensitivity was 20.91 nm/g at room temperature. At 600 °C, the sensitivity of the sensor was affected by temperature decreases to 15.6 nm/g. 

Based on the above analysis, it can be seen that, in terms of sensor manufacturing methods, laser processing methods have a high processing accuracy. Most laser processing tends to fabricate miniature structures on the fiber optic in order to take full advantage of the high precision. Due to the special shape of the fiber optic, these sensors usually have a small measurement bandwidth and vibration sensors made are mainly used in specific fields. MEMS technology is one of the potential technologies to realize sensor fabrication and batch production with the advantages of high reliability and small size [27]. Compared to the above processing methods, MEMS technology is planarized processing, which is highly accurate and simple to control. Therefore, the material and process used in the sensor restrict the working temperature, batch production, and consistency of the sensor, and the measurement accuracy of the sensor is affected by temperature. The combination of the sensing technology and MEMS technology provides a reliable solution for the miniaturization and batch production of vibration sensor in high-temperature environments. In our previous studies, we proposed a vibration sensor based on silicon material, with a working temperature of 400 °C [28]. By optimizing the material composition and preparation process of the sensor sensitive unit, the working temperature of the sensor was improved to 800 °C [29].

In this paper, a fiber-optic FP sensor based on single-crystal magnesium oxide (MgO) is proposed for vibration measurement at 1000 °C. The sensor is fabricated by developing MgO MEMS technology, utilizing wet chemical etching and direct bonding for consistent manufacturing and batched manufacturing, thus achieving the all-MgO vibration sensor. The four-sided fixed beam structure increases the resonant frequency of the sensor, resulting in a larger operating bandwidth and making the sensor work more stably in noisy environments. Using the three-wavelength demodulation method combined with a temperature-decoupling algorithm to demodulate the vibration signal, the temperature–vibration cross-sensitivity is reduced and the testing accuracy is improved. This sensor is expected to meet the vibration parameter measurement requirements of aero-engines and gas turbines in harsh environments.

## 2. Sensor Structure and Sensing Principle

**Figure 1:**
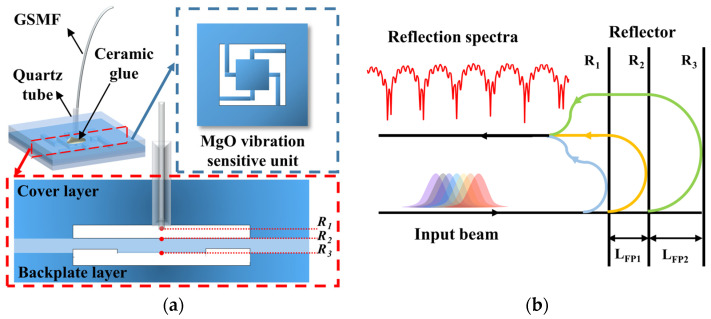
(**a**) The schematic diagram for the vibration sensor structure. (**b**) Hybrid cavities interference model of the sensor.

The schematic diagram for the vibration sensor structure is shown in Figure 1a. The sensor mainly consists of a sensor head, a gold-plated single-mode fiber (GSMF, Fiber guide, Stirling, NJ, USA), and a quartz tube. The sensor head consists of three layers of MgO bonded directly together. The first layer (cover layer) has a through hole and a countersunk hole, which are used to fix the collimated structure of the quartz tube and GSMF. The second layer is a vibration-sensitive unit, which is graphically etched by wet-etching technology to obtain a square mass block and cross-beam structure. When an excitation is generated from outside to act on the sensor, the mass block and cross beams of the sensor deform as a sensitive structure so that it can sense the acceleration change from outside. When the temperature changes, the thickness of the mass block will change due to the thermal expansion effect of the MgO material. By monitoring the change in thickness, the ambient temperature can be obtained. The third layer (backplate layer) has a countersunk hole, which is used to reserve space for the up and down displacements of the beam–mass block structure. The countersunk holes and through holes in the backplate and cover layers were obtained by mechanical processing, which can make the processed countersunk holes have a high roughness and reduce their light reflection. 

The sensor is a multi-cavity fiber-optic extrinsic FP interferometer (EFPI) vibration sensor, which contains three reflective surfaces. The hybrid cavities interference model of the sensor is shown in Figure 1b. R_1_ denotes the fiber-optic end face. R_2_ and R_3_ denote the upper and bottom surfaces of the mass block, respectively. The FP_1_ cavity composed of R_1_ and R_2_ is used for measuring the vibration, with the length of $L_{{\mathrm{FP}}_{1}}$. The FP_2_ cavity composed of R_2_ and R_3_ is used for measuring the temperature, with the length of $L_{{\mathrm{FP}}_{2}}$. The light beams emitted by the light source are reflected by the three reflective surfaces and go back into the fiber core, forming the interference signal. The interference signal is expressed as

(1) $$I_{r}=I_{1}{+I}_{2}+I_{3}+2\sqrt{I_{1}I_{2}}\cos\theta_{1}+2\sqrt{I_{2}I_{3}}\cos\theta_{2}+2\sqrt{I_{1}I_{2}}\cos{(\theta }_{1}+\theta_{2}),$$

where $I_{r}$ represents the intensity of the reflected light. $I_{1}$, $I_{2}$, and $I_{3}$ represent the reflected light of the three reflective surfaces. $\theta_{1}=4\pi n_{1}L_{{\mathrm{FP}}_{1}}/\lambda$ and $\theta_{2}=4\pi n_{2}L_{{\mathrm{FP}}_{2}}/\lambda$ are the interference phases generated by light reflected by the up and down surfaces of FP_1_ and FP_2_, respectively. $n_{1}$ and $n_{2}$ are the refractive indices of air and MgO.

When the external acceleration excitation is applied to the sensor, the distance between the mass block and the fiber end face changes for the deformation of the beam structure. Therefore, $L_{{\mathrm{FP}}_{1}}$ changes and the phase of the interference signal changes. The external acceleration can be obtained by converting the demodulated phase change of the interference signal. The relationship between the phase variation of the interference signal $∆\theta_{1}$ and the $L_{{\mathrm{FP}}_{1}}$ variation $∆L_{{\mathrm{FP}}_{1}}$ is expressed as

(2) $$∆\theta_{1}=(4\pi n_{1}/\lambda )∆L_{{\mathrm{FP}}_{1}},$$

where λ represents the wavelength of light.

According to Newton’s Second Law and Euler–Bernoulli beam theory, the maximum deflection of the cross beam when axial acceleration excitation is applied to the above system $\Delta Y_{\max}$ is

(3) $$∆Y_{\max}=-ml^{3}a/4\mathrm{Eb}h^{3},$$

where m represents the mass of the mass block, l, b, and h represent the length, width, and thickness of a single beam, respectively, E represents the Young’s modulus of MgO, and a represents the axial acceleration excitation. The axial displacement of mass block $∆L_{{\mathrm{FP}}_{1}}$ caused by axial acceleration is numerically equivalent to the maximum deflection of the cross beam $∆Y_{\max}$. 

The sensitivity in this paper denotes the displacement value of the sensor mass block under 1 g acceleration, which can also be regarded as the cavity length change in FP_1_ of the sensor. The sensitivity S of the sensor is expressed as

(4) $$S=\mathrm{mg}l^{3}/4\mathrm{Eb}h^{3}.$$

The resonant frequency f of the sensor is expressed as

(5) $$f=1/2\pi \sqrt{K/m}=1/\pi \sqrt{\mathrm{Eb}h^{3}/ml^{3}},$$

where $K=\mathrm{ma}/∆Y_{\max}$ represents the stiffness of the beam–mass block structure [30]. According to Equations (4) and (5), the sensitivity and the resonance frequency of the sensor change inversely with the variation of dimension of the beam and the mass block. Therefore, the ideal sensitivity and resonance frequency can be obtained by flexibly designing the length, width, and thickness of the beam and mass block.

In the sensitive unit structure designed for this sensor, the beam length is 3.8 mm, the beam width is 0.3 mm, and the side length of the mass block is 4 mm. According to Equations (4) and (5), the theoretical sensitivity and resonant frequency of the sensor are 0.9207 nm and 15,915 Hz, respectively.

## 3. Sensor Fabrication

**Figure 2:**
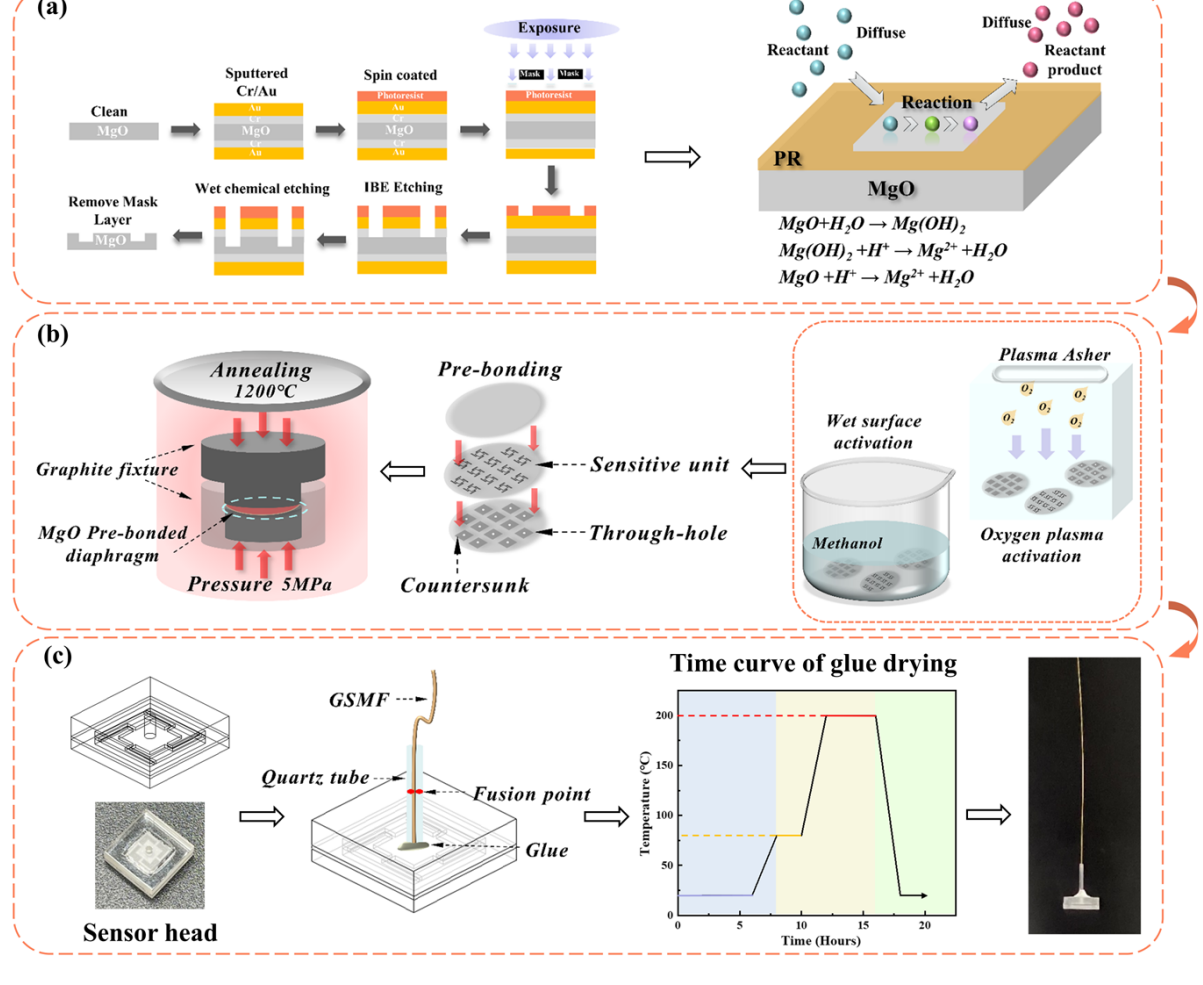
The schematic diagram of the preparation process of the sensor. (**a**) Sensitive unit preparation by wet chemical etching. (**b**) Direct bonding of three-layer MgO wafers. (**c**) Sensor manufacturing process and physical image.

Double-sided polished MgO wafers with {100}-faces were used as the sensitive unit layer, cover layer, and backplate layer of the sensor head. The fabrication process of this sensor included the sensitive unit preparation by wet chemical etching, the direct bonding of the three-layer MgO wafers, and the integration of the fiber optic and sensor head, as shown in Figure 2.

**Figure 3:**
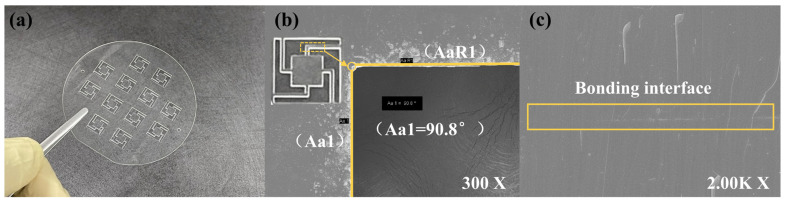
The morphology characterization results after etching. (**a**) Etched sensitive element array. (**b**) SEM image of the etched boundary. (**c**) SEM image of the bonding interface at 2.00K X magnification.

The high hardness and brittleness of MgO make it difficult to process. Based on previous research [31], the wet etching technology is optimized to efficiently and accurately fabricate mass blocks and beam structures, as shown in Figure 2a. The MgO wafer was cleaned sequentially with gasoline, alcohol, SC-1 solution (NH_4_OH:H_2_O_2_:H_2_O = 1:2:7), and deionized water, and then was blown dry with nitrogen gas (N_2_). After cleaning, residual organic matter and impurities on the surface could be removed. Gold and chromium were selected as mask layers for MgO wet chemical etching because of their high etch selectivity ratio, chemical stability, and tight bonding with the MgO surface layer after magnetron sputtering. The transfer of beam and mass block patterns were achieved using the standard photolithography technology. The metal mask layer exposed after photolithography and development was etched using IBE etching, and the etching gas used was argon gas. Due to the significant difference in size between the beam structure and the mass block structure, there are high requirements for the etching accuracy and rate. Therefore, high-concentration phosphoric acid etchants were selected for graphical array fabricating. Based on the characterization of the structure size and etching morphology after etching, the final etchant was determined to be 80% phosphoric acid (H_3_PO_4_) at a temperature of 80 °C. Figure 3 shows the morphology characterization results after etching. Figure 3a shows multiple vibration sensor sensitive units after the wet etching of MgO, and Figure 3b shows the image of the etched MgO edges under Scanning Electron Microscope (SEM, HITACHI, Tokyo, Japan, SU 5000). It can be seen that the etching edges are smooth and continuous, and the angle is close to a right angle at the turning position of the structure. The etching condition realizes a high precision, large-size-ratio patterning, and high consistency in the processing of sensitive structures.

The hydrophilic direct bonding of MgO/MgO method was used to realize the thermal stress matching of the MgO cover layer, the etched vibration sensitive unit, and the backplate layer [32], as shown in Figure 2b. Specifically, the etched vibration sensitive unit and the other two MgO layers were cleaned. Oxygen plasma surface activation and wet chemical surface activation were carried out using Plasma Asher (PVA TePla, Wettenberg, Germany, IoN 10) and SC-1 solution (NH_4_OH:H_2_O_2_:H_2_O = 1:1:5), respectively. Through these operations, the surface energy and hydrophilicity of the wafer were improved. Pre-bonding was carried out in the air environment of a thousand-level clean room. Subsequently, the pre-bonded structure was transferred to a high-temperature hot pressing furnace for the high-temperature annealing treatment, with an annealing pressure of 5 MPa and an annealing temperature of 1200 °C. The SEM testing was conducted on the bonding interface, as shown in Figure 3c. It can be seen that the MgO bonding interface is tight, ensuring the structural stability of the sensor in high-temperature impact environments. At this point, the preparation of the MgO sensor head was completed.

Finally, the sensor head and fiber optic were integrated, as shown in Figure 2c. The GSMF was cut flat and inserted into the quartz tube. They were welded using a CO_2_ laser. The quartz tube was then inserted into the through hole in the cover layer of the sensor head and fixed by a high-temperature-resistant adhesive (Yikun, Hubei, China, YK–8927). The adhesive was dried after fixation according to the curve shown in the Figure 2c. The main components of the adhesive are refractory ceramics such as silica-aluminate and inorganic polymers, with a heat-resistant temperature of 1300 °C and a coefficient of linear expansion of 12.0 × 10^−6^/°C, similar to that of MgO (11.2 × 10^−6^/°C).

## 4. Testing and Discussion

**Figure 4:**
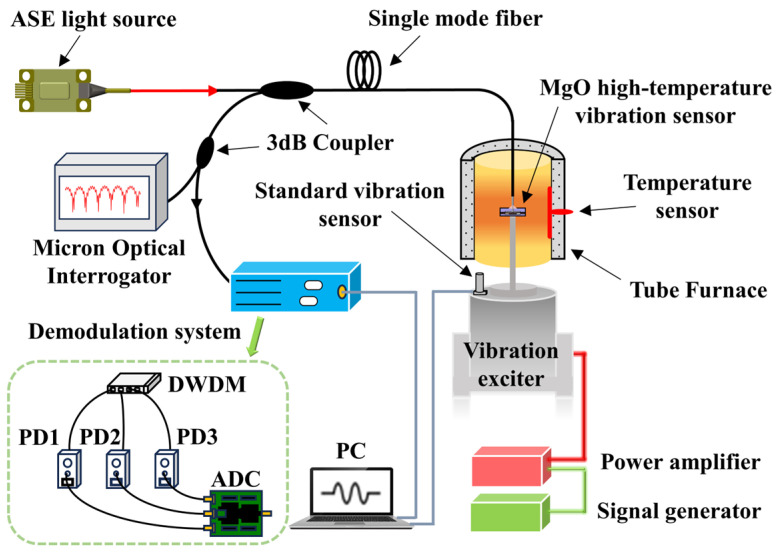
High-temperature vibration composite test system.

To realize the specific FP cavity dynamic demodulating of the multi-cavity EFPI sensor, a self-compensated three-wavelength demodulation method was adopted and optimized [33]. A high-temperature vibration testing system was established for investigating the acceleration response of this sensor under different temperature conditions, as shown in Figure 4. A flat-top amplified spontaneous emission (ASE) light source was used, with the wavelength range of 1525–1600 nm. Light from the ASE light source was transmitted to the vibration sensor through a single-mode fiber and a 3 dB fiber-optic coupler. The light reflected from the sensor was split into three different wavelengths by dense wavelength division multiplexing. Three interferometric signals at each center wavelength were obtained by three photodiodes (PDs): PD_1_, PD_2_, and PD_3_.The voltage signals were collected by an analog-to-digital conversion system and transmitted to a computer. The length of the cavity FP_1_ was 91.036 µm for the sensor used in the experiment. According to Equation (2), the output of this demodulation system is the amount of phase change $\Delta \theta_{1}$, which can be expressed as

(6) $$\Delta \theta_{1}=(4\pi /\lambda_{C})∆L_{{\mathrm{FP}}_{1}},$$

where $\;\lambda_{C}$ represents the average value of the three wavelengths used in the demodulation system. Based on the Equation (6), when the acceleration excitation is 1 g, the calculated $∆L_{{\mathrm{FP}}_{1}}$ can be regarded as the sensitivity of the sensor. Therefore, the theoretical sensitivity of 0.9207 nm calculated by Equation (4) can be rewritten as 0.00737 rad.

A standard accelerometer was fixed on the surface of the vibration exciter (TIRA, Schalkau, Germany, TV 50101) and the MgO fiber-optic vibration sensor was fixed on the vibration exciter through connecting rods. The fiber-optic sensor was placed in the constant temperature zone of the tube furnace (HF Kejing, Hefei, China, OTF–1200X), which is equipped with a temperature sensor to provide real-time temperature. In this system, the standard accelerometer was used to calibrate the acceleration value applied on the fiber-optic sensor. The acceleration signal of the fiber-optic vibration sensor was obtained through a demodulation system, and the spectrum of the fiber-optic vibration sensor at different temperatures were obtained through a Micron Optical Interrogator (MOI, Micron Optics, Atlanta, GA, USA, SM–125).

**Figure 5:**
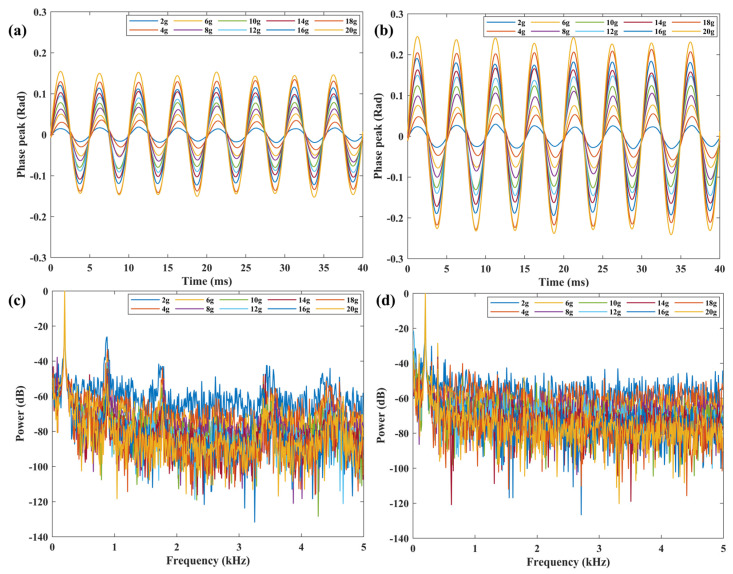
Time–domain signal at (**a**) 20 °C, and (**b**) 1000 °C. Frequency-domain signal at (**c**) 20 °C, and (**d**) 1000 °C.

The sensor was evaluated over a temperature range of 20–1000 °C in increments of 200 °C. At each temperature, the sensor was tested between 2–20 g in increments of 2 g. The excitation frequency was 200 Hz. During vibration testing, in order to accurately record the output of each acceleration point, hold each acceleration point for 1 min when it is reached before collecting data. The responses of the vibration sensor are displayed in Figure 5a,b, showing the time-domain signals at 20 °C and 1000 °C, respectively. It can be seen that the output-phase peaks of the sensor at the same temperature increases with the rise in acceleration. At the same acceleration, the output-phase peaks of the sensor increase with the rise in temperature. The sensitivity of the system reaches 0.0073 rad and 0.012 rad at the temperature of 20 °C and 1000 °C, respectively. Figure 5c,d show the frequency spectrum obtained by fast Fourier transform corresponding of the signals in Figure 5a,b. It can be seen that the demodulated signal frequency is 200 Hz, which is consistent with the frequency of the excitation.

**Figure 6:**
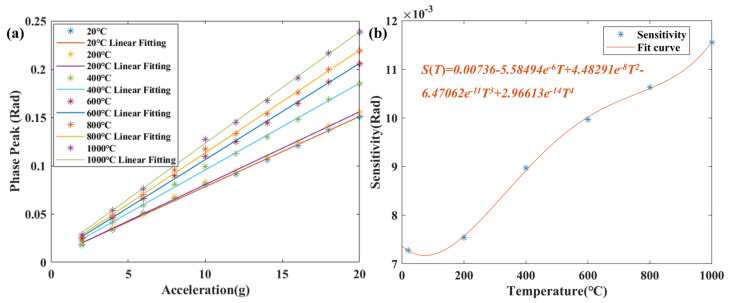
(**a**) Vibration test within the range of 20–1000 °C. (**b**) The fitting curve between sensor sensitivity and temperature.

In order to further analyze the temperature cross-sensitivity of the sensor, the output peaks of the sensor at 20–1000 °C under different accelerations were recorded. The demodulated results at different temperatures are shown as a function of acceleration, as shown in Figure 6a. It can be seen that there is a linear relationship between the sensor output and the measured acceleration value at each temperature, indicating that the sensor with MgO maintains the characteristics of a second-order system below 1000 °C. This is attributed to the MgO direct bonding technology without an intermediate transition layer and the integrated processing scheme. Figure 6b shows that the slope of the linear fit of the sensor output increases with the rise in temperature, which means that the sensitivity of the sensor increases with the rise in temperature. This is due to the effect of temperature on mechanical parameters such as the coefficient of thermal expansion and Young’s modulus of the MgO. Therefore, according to the temperature-coupling characteristics of the sensor, the decoupling is carried out to correct the temperature cross-sensitivity, so as to improve the measurement accuracy of the sensor.

**Figure 7:**
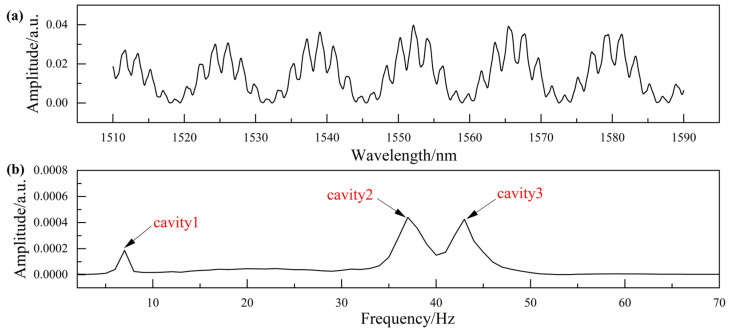
Signal demodulation. (**a**) Reflection spectrum of the sensor. (**b**) Frequency spectrum.

The EFPI interference signal of the sensor was collected by MOI, as shown in Figure 7a. The thickness of the mass block will change with the different ambient temperature of the sensor, which is reflected in the small envelope change of the spectrum signal. Therefore, it is necessary to separately extract the spectrum of the mass block to decouple the temperature of the sensor. Figure 7b shows the Fourier-transform frequency spectrum of the interference signal. Three peaks correspond to the frequencies of the interference signals formed by R_1_ and R_2_ (cavity 1), R_2_ and R_3_ (cavity 2), and R_1_ and R_3_ (cavity 3) in the sensor, respectively. 

Based on the spectrum pattern of an ideal FP sensor, a simulated spectrum $S_{v}\left(\lambda \right)$ is constructed. Then, using the software cross-correlation algorithm, the correlation number C between the real spectrum and the virtual spectrum obtained is expressed as [34]

(7) $${C(d}_{v})=\sum_{n=1}^{n=N}S\left(\lambda \right)S_{v}\left(\lambda \right)=\sum_{n=1}^{n=N}S(\lambda )\cos(4\pi d_{v}/\lambda_{n}+\pi ),$$

where $d_{v}$ represents the simulated cavity length, $\lambda_{n}$ represents the wavelength, and N represents the number of the spectrum sampling points. The cavity length corresponding to the maximum value of the cross-correlation coefficient denotes $L_{{\mathrm{FP}}_{2}}$.

Once the sensor spectrums and the responses of the sensor are obtained in calibration testing at different temperatures, the software cross-correlation algorithm can be applied to calculate $L_{{\mathrm{FP}}_{2}}\left(T\right)$ at different temperatures. And the vibration sensitivity $S\left(T\right)$ of the sensor at different temperatures can be computed by the self-compensated three-wavelength demodulation method. When the sensor works at an unknown temperature, the temperature $T_{n}$ can be determined by $L_{{\mathrm{FP}}_{2}}\left(T\right)$. Then, substitute $T_{n}$ into $S\left(T\right)$ to obtain $S_{n}$. As a result, the decoupled value of the acceleration signal a is expressed as

(8) $$a=\varphi_{T}/S_{n},$$

where $\varphi_{T}$ represents the response of the sensor to external acceleration at $T_{n}$.

**Figure 8:**
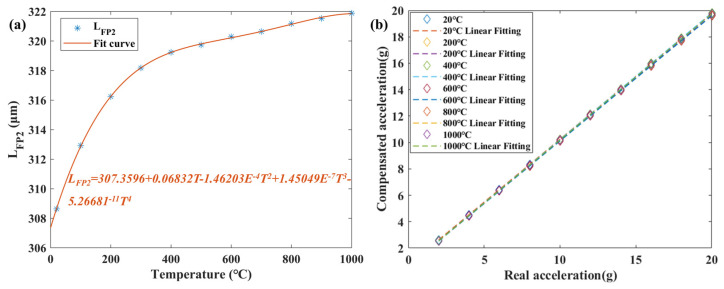
(**a**) L_FP_2__ versus temperature. (**b**) Temperature decoupling results from 20–1000 °C.

Based on the above principles, $L_{{\mathrm{FP}}_{2}}$ was calculated based on the spectra obtained from the MOI at 20–1000 °C, as shown in Figure 8a. The calculated data points were fitted and the curve fit was 99.978%. By using the above temperature-decoupling method, the comparison between the decoupled acceleration values at each temperature and the actual acceleration values is obtained, as shown in Figure 8b. It can be seen that the calculated values at each temperature are in good agreement with the actual acceleration values of the vibration exciter, and the maximum nonlinearity error is 1.17%.

## 5. Conclusions

In this paper, a high-temperature fiber-optic vibration sensor based on MgO has been demonstrated. By optimizing the MEMS processing technology of MgO, the high-precision and high-quality processing of the sensor’s sensitive unit is achieved, which increases the maximum working temperature of the sensor. The batch manufacturing process helps to maintain a consistent sensor performance, and the high production rates offer the potential for further commercialized applications. The results of high-temperature vibration experiments show that the sensor can operate at 1000 °C, and the sensor shows a nearly linear response in the acceleration range of 2–20 g at different temperatures. The occurrence of FP interference in multiple cavities allows the sensor to obtain temperature and vibration parameters, thereby achieving temperature decoupling. The maximum nonlinearity error of the vibration sensor measurement after temperature decoupling is 1.17%. Compared with conventional sensors, the MgO fiber-optic vibration sensor described in this paper solves many problems that limit the application of other vibration sensors, such as the thermal damage to sensitive materials, thermal stress mismatch, adhesive rupture, and sensor performance changes caused by temperature changes. The processing and fabrication methods for MgO fiber-optic sensors demonstrated in this work can be extended to the development of multiparameter measurement sensors. The proposed temperature decoupling method can provide a reference for the measurement of other mechanical parameters at high temperature to improve the test accuracy. In addition, the working temperature of the sensor can be increased by studying the transmission method of the signal in future work.

## Data Availability

The data will be made available upon request.
